# Pyridinium based amphiphilic hydrogelators as potential antibacterial agents

**DOI:** 10.3762/bjoc.6.101

**Published:** 2010-09-21

**Authors:** Sayanti Brahmachari, Sisir Debnath, Sounak Dutta, Prasanta Kumar Das

**Affiliations:** 1 Department of Biological Chemistry, Indian Association for the Cultivation of Science, Jadavpur, Kolkata – 700 032, India. Fax: +(91)-33-24732805

**Keywords:** antibacterial, bilayer structure, hydrogel, pyridinium, self-assembly

## Abstract

The numerous applications of hydrogelators have led to rapid expansion of this field. In the present work we report the facile synthesis of amphiphilic hydrogelators having a quaternary pyridinium unit coupled to a hydrophobic long alkyl chain through an amide bond. Different amphiphiles with various hydrophobic chain length and polar head groups were rationally designed and synthesized to develop a structure-property relation. A judicious combination of hydrophilic and hydrophobic segments led to the development of pyridinium based amphiphilic hydrogelators having a minimum gelation concentration of 1.7%, w/v. Field emission scanning electronic microscopy (FESEM), atomic force microscopy (AFM), photoluminescence, FTIR studies, X-ray diffraction (XRD) and 2D NOESY experiments were carried out to elucidate the different non-covalent interactions responsible for the self-assembled gelation. The formation of three-dimensional supramolecular aggregates originates from the interdigitated bilayer packing of the amphiphile leading to the development of an efficient hydrogel. Interestingly, the presence of the pyridinium scaffold along with the long alkyl chain render these amphiphiles inherently antibacterial. The amphiphilic hydrogelators exhibited high antibacterial activity against both Gram-positive and Gram-negative bacteria with minimum inhibitory concentration (MIC) values as low as 0.4 μg/mL. Cytotoxicity tests using MTT assay showed 50% NIH3T3 cell viability with hydrogelating amphiphile **2** up to 100 μg/mL.

## Introduction

Gels are an outstanding group of soft materials lying at the interface of solid and liquid, and find numerous applications in various fields including tissue engineering, biosensors, food processing, cosmetics, photography, controlled drug delivery etc. [1–11]. Amongst the variety of gels, hydrogels (those that entrap water) are of special importance owing to their tremendous potential in biomedicine [12–16]. These hydrogels can be of natural origin [17] (collagens, polysaccharides) as well as of synthetic origin [18] (poly(acrylic acid) and derivatives, polypeptides and small molecules). This fascinating class of materials results from the spontaneous self-assembly of polymeric/non-polymeric molecules that lead to the formation of supramolecular three dimensional (3D) networks with interstitial space for the immobilization of solvents. Low-molecular-weight-gelators (LMWG) have received more attention than their polymeric analogues for a number of scientific applications due to their thermo-reversible nature and their prompt response to external stimuli [19]. A critical balance between hydrophilic and hydrophobic interactions is mandatory for any gelation process. Non-covalent interactions such as hydrogen bonding, ionic interactions, π–π stacking or van der Waals forces play a pivotal role in self-assembled gelation [12]. Tuning the structure of gelator molecules leads to a better understanding of the contribution of the different interactive forces and an insight into the 3D-morphology of supramolecular aggregates [20–22]. In this context, low molecular weight hydrogels (LMWH) are of greater importance compared to polymeric ones as the former (i) can have diversified supramolecular morphology by varying the structure of its precursor molecules, (ii) have the ability for quick response to external stimuli, and (iii) are potentially biocompatible [12,23–24]. Thus, the huge range of applications of the hydrogels and the beneficial aspects of small molecule gelators including ease of preparation have synergistically led to a surge in the development of tailor-made LMWHs.

The presence of an aromatic ring (for example, phenyl, naphthalene, *N*-fluorenyl-9-methoxycarbonyl (Fmoc), indole, pyridine) in small molecule gelator is known to have crucial influence in inducing self-aggregation towards gelation. The planar aromatic moiety favors π–π stacking interactions between the molecules and leads to the formation of 3D networks of viscoelastic gels [25–27]. Interestingly, among all these aromatic rings, the positively charged pyridine (pyridinium) unit is well known to impart antibacterial properties to amphiphilic molecules [28–31]. The cationic charge of the amphiphile plays an instrumental role in disrupting the innate defense mechanism of microorganisms by disrupting the microbial cell membrane [32–33]. Hence, it would be interesting to develop amphiphilic hydrogelators that have pyridinium moieties in order to exploit its favorable π–π stacking interaction towards self-assembled gelation as well as an ability to kill bacteria. Furthermore, a very simple method of synthesizing such amphiphilic antibacterial hydrogelators with pyridinium units would definitely boost its importance and utility for a wide spectrum of applications.

**Figure 1:**
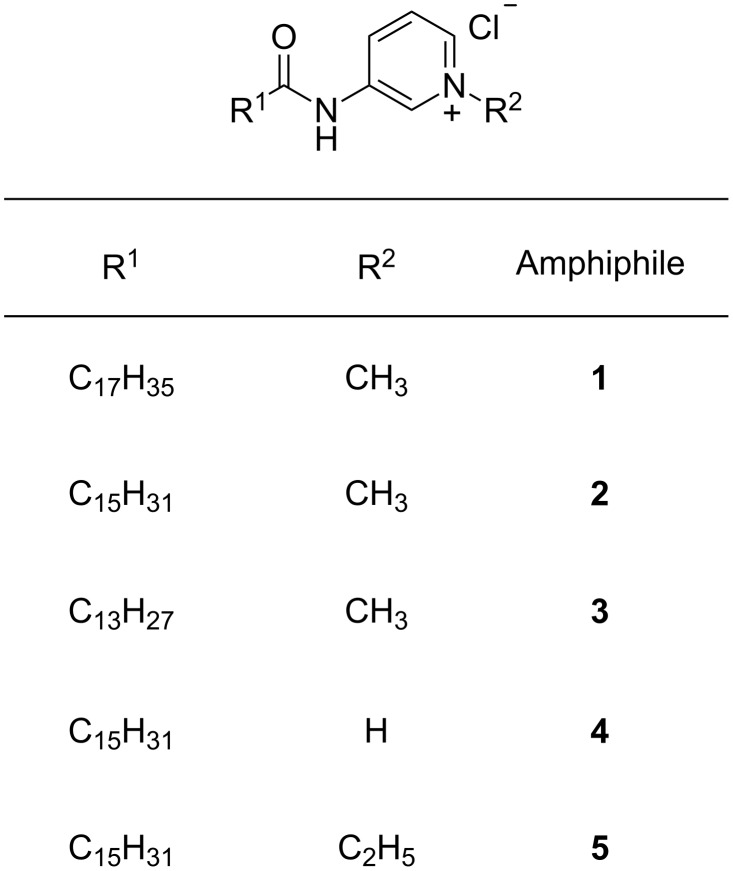
Structure of amphiphiles **1**–**5**.

In the present work, we report the facile synthesis of pyridinium based amphiphiles (**1**–**5**, Figure 1) of which amphiphiles **1** and **2** were efficient hydrogelators with minimum gelation concentrations (MGC) ≈1.7–2.0%, w/v. Modification of certain features of the amphiphiles such as the aliphatic chain length and the polar head group was systematically carried out to understand their influence on the self-assembled hydrogelation. The various factors involved in the formation of supramolecular aggregates leading to hydrogelation were studied using FTIR, XRD and fluorescence spectroscopy. The topographical features of the soft matter were visualized using different microscopic techniques (scanning electron microscopy (SEM), atomic force microscopy (AFM)). Interestingly, these compounds were found to show excellent antibacterial activity against Gram-positive and Gram-negative bacteria with minimum inhibitory concentration (MIC) values as low as 0.4 µg/mL for *Micrococcus luteus*. In addition, amphiphile **2** was investigated for cytotoxicity with mammalian cells (NIH3T3) and showed sufficient viability throughout a range of concentrations.

## Results and Discussion

**Scheme 1:**
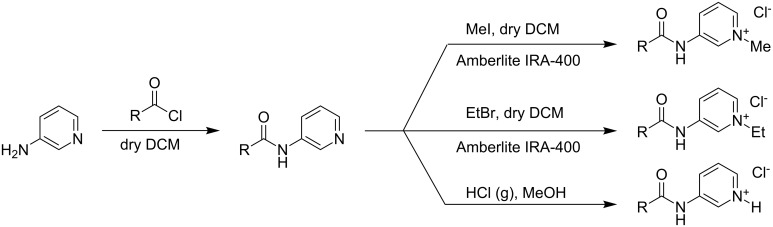
Synthetic procedure of the amphiphiles.

Gelation is simply a macroscopic manifestation of the self-assembled aggregation at the molecular level due to the optimum combination of hydrophilic and hydrophobic interactions between molecules [12]. The formation, nature and morphology of these supramolecular 3D-networks are primarily dictated by the architecture of the gelating molecules. To establish the different nature of interactions taking place within the supramolecular assemblies, a structure-property correlation for the gelators is necessary. In the present work we have synthesized a series of amphiphilic compounds containing a quaternary pyridinium unit as the polar head group and varied the length of alkyl chain of the hydrophobic part (**1**–**5**, Figure 1) using very simple methodology (Scheme 1). Variation at the hydrophilic as well as the hydrophobic segment was performed to understand the critical balance imperative for hydrogelation.

Amphiphile **1**, with a *N*-methylated pyridinium moiety as the polar head linked by a C-18 alkyl chain through an amide bond, exhibited efficient water gelation ability (MGC of 2.0%, w/v). The stable to inversion of container method confirmed the formation of gel. However, the hydrogel was not stable as the amphiphile precipitated from the gel after 4–5 h. It is possible that the C-18 alkyl chain is too hydrophobic to maintain the optimum hydrophilic–hydrophobic balance necessary for efficient gelation. Consequently, keeping all other segments identical as in **1**, the C-18 alkyl chain was replaced by a shorter alkyl chain, C-16 in case of amphiphile **2**. In accord with our expectations, amphiphile **2** exhibited better water gelation ability with a MGC of 1.7%, w/v. The transparent hydrogel of **2** was stable for several months. At this point, we were curious to know how the gelation efficiency would be affected by further lowering the alkyl chain length to C-14 (amphiphile **3**). However, compound **3** was found to be a non-gelator. A decrease in the hydrophobicity in amphiphile **3** possibly destroyed the hydrophilic–hydrophobic balance required for water gelation. Following the importance of the hydrophobic segment of the amphiphile in gelation, we were also interested to investigate the influence of the polar head group of the pyridinium-based amphiphiles in hydrogelation. To this end we made very minor modifications to the quaternized nitrogen of the pyridine moiety keeping other segments unaltered as in the efficient gelator **2**. *N*-methylated pyridinium of **2** was changed first of all to a simple protonated pyridinium moiety in amphiphile **4** and then to *N*-ethylated pyridinium group in the case of **5** (Figure 1). Neither of these amphiphiles exhibited any water gelation ability, which reiterates the importance of the optimum balance between hydrophilic and hydrophobic character within a molecule for gelation.

**Figure 2:**
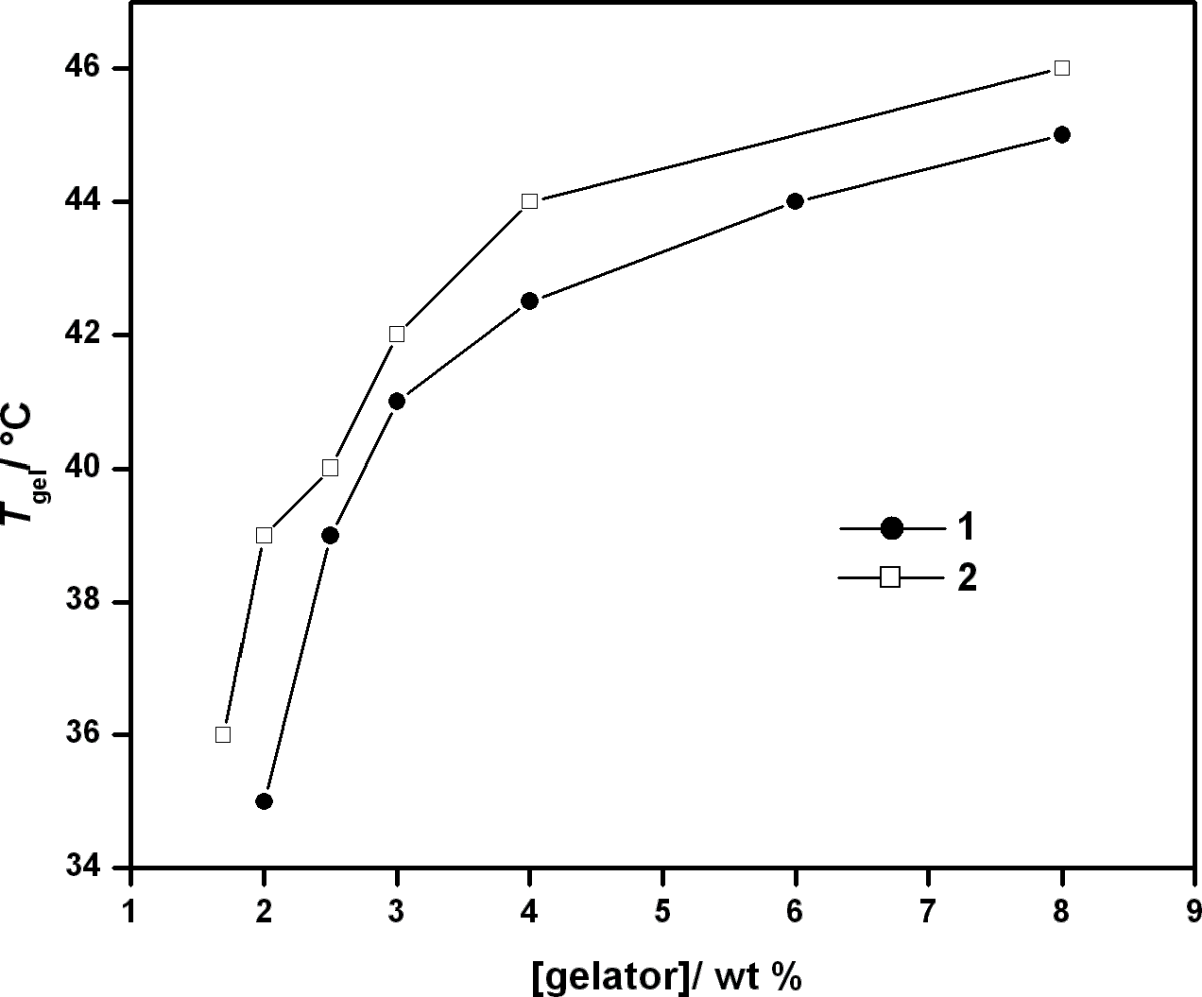
Variation of the *T*_gel_ with concentration of amphiphiles **1** and **2**.

The gel-to-sol transition temperature (*T*_gel_) for both the hydrogels was determined by placing the gel-containing glass vial (inner diameter = 10 mm) in a thermostated oil bath and raising the temperature slowly at a rate of 2 °C/min. *T*_gel_ is defined as the temperature (±0.5 °C) at which the gel melts and starts to flow from an inverted glass vial. Both hydrogels of **1** and **2** at their MGC showed *T*_gel_ at 35 and 36 °C, respectively. In agreement with the previous reports it was found that the *T*_gel_ of the hydrogels steadily increased with increase in gelator concentration (Figure 2) [34–35]. This clearly indicates the enhancement in the strength of the non-covalent intermolecular interaction in the aggregated state with increasing gelator concentration. Also the thermo-reversible nature of hydrogelation was established as the sol formed on heating returned to the gel state upon lowering the temperature. Interestingly, the *T*_gel_ curve of amphiphile **2** maintained a slightly higher profile throughout the range of the concentrations suggesting the comparatively better hydrogelation efficiency of **2** over that of **1**.

**Figure 3:**
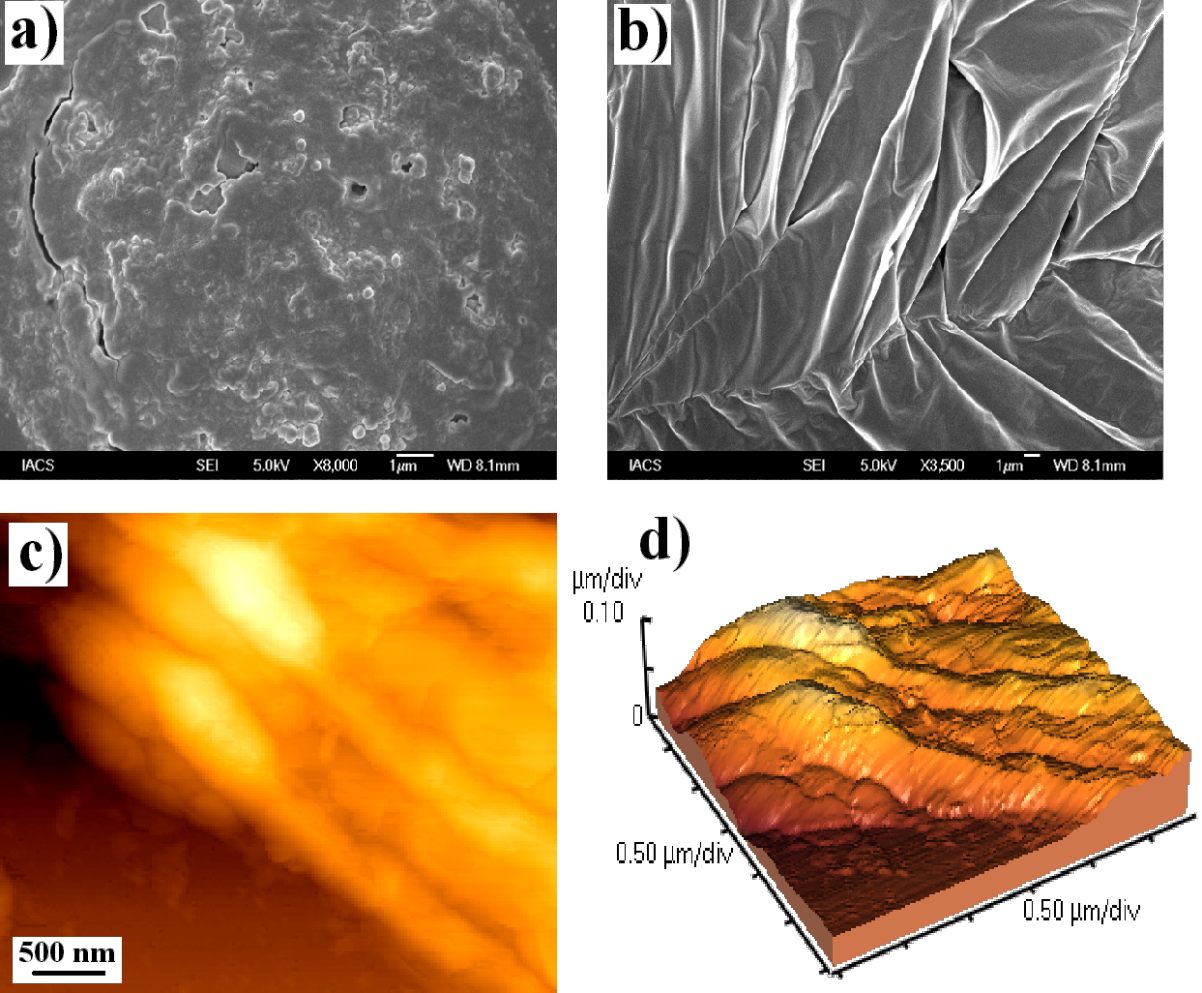
(a, b) FESEM images of the dried gels of **1** and **2**, respectively at their MGC. (c, d) Two- and three-dimensional AFM image of xerogel **2**.

The formation of three dimensional higher ordered structures during self-assembled hydrogelation was investigated by field emission scanning electron microscopy (FESEM). Morphology of the dried xerogels showed the formation of different supramolecular structures that are involved in the gelation process of **1** and **2** (Figure 3). SEM image of hydrogel **1** showed an aggregated form of porous networks (Figure 3a), which were responsible for the entrapment of the solvent. In case of hydrogel **2**, formation of thin intertwined fibrillar networks of 200–300 nm dimensions (Figure 3b) was observed. The fibrillar architecture of **2** at the aggregated state was further confirmed by Atomic Force Microscopic (AFM) images. Two and three dimensional AFM images of xerogel **2** (Figure 3c, d) showed the involvement of fibrillar networks in self-assembled hydrogelation. The dimension of the fibril network observed in the AFM image was also in accord with the FESEM images.

**Figure 4:**
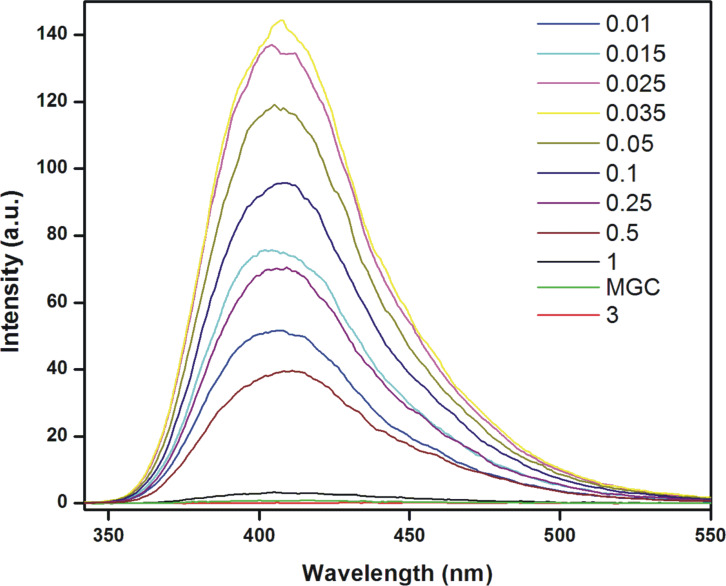
Luminescence spectra of **2** in water (λ_ex_ = 330 nm) at various concentrations and room temperature.

The participation of the pyridinium ring in hydrogelation was investigated by taking the luminescence spectra of the efficient gelator **2** at various concentrations (0.01–3.0%, w/v) in water (Figure 4) at room temperature. The amphiphile **2** was excited at λ = 330 nm and the emission spectra were recorded between of 340–550 nm. At a very low concentrations (0.01%, w/v), **2** showed an emission peak at λ_em_ = 402 nm. With a gradual increase in the concentration of **2**, a steady increase in the fluorescence intensity was observed up to 0.035%, w/v. With further increase in the concentration of **2**, the fluorescence intensity decreased with a continuous red shift of the λ_em_ from 402 nm to 416 nm_._ The observed increase in the fluorescence intensity as well as continuous quenching of the signal after 0.035%, w/v to MGC and above is probably due to the interaction between the pyridinium ring and the cationic charge (Figure 4) [36]. Notably, the quenching in the emission of pyridine started at a concentration that is ≈50 times lower than the corresponding MGC which is also almost three times higher than its critical micellar concentration (0.011%, w/v). Hence, the amphiphile **2** began to self-assemble towards hydrogelation above 0.035%, w/v. Moreover, the red shifted emission peak up to MGC and above indicates that the intermolecular π–π interactions between the pyridine moieties plays an important role in gelation [28]. Consequently, the fluorescence quenching of pyridine by the cationic charge was due to the close proximity of the head groups during gelation.

**Figure 5:**
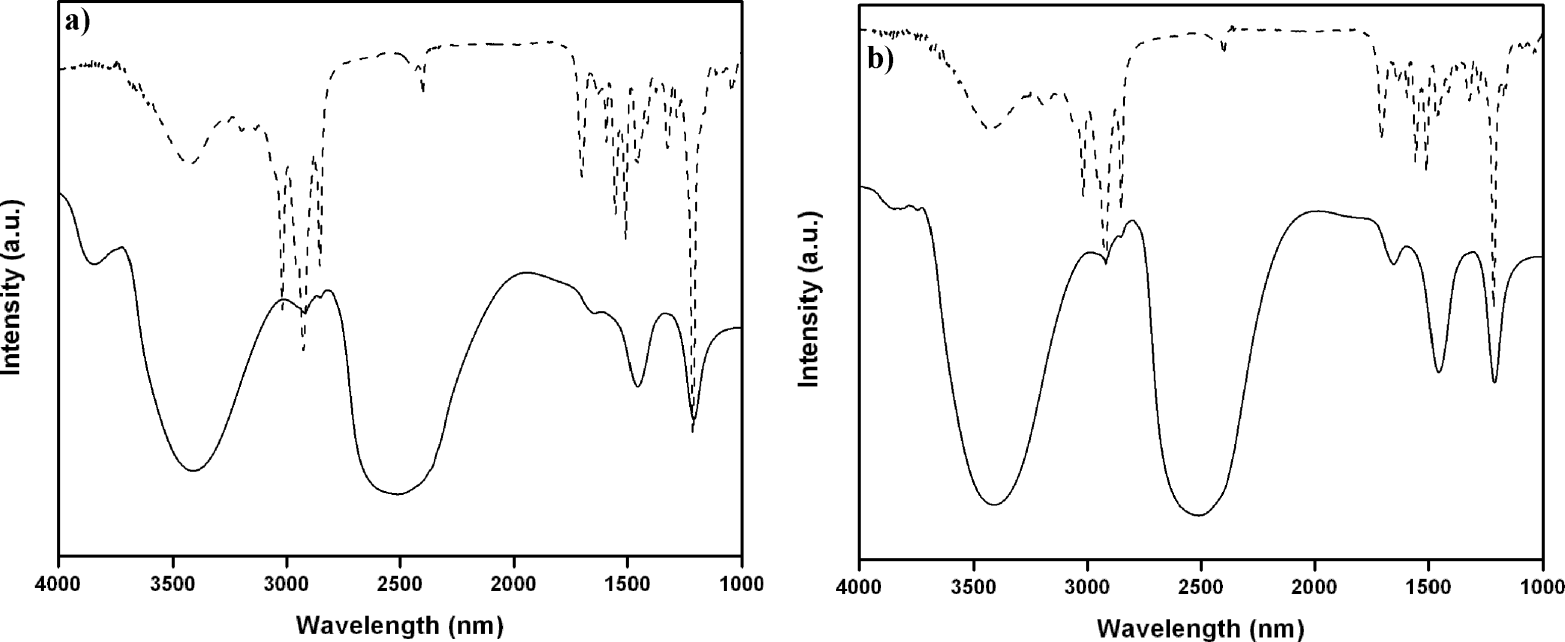
FTIR spectra of (a) **1** and (b) **2** in CHCl_3_ solution (dashed line) and in D_2_O at the gel state (solid line).

To determine the involvement of intermolecular hydrogen bonding between the amide N–H and carbonyl oxygen we investigated both hydrogels by FTIR spectroscopy. As the presence of H_2_O in FTIR spectroscopy may create difficulties in extracting information on intermolecular interactions, we measured the FTIR spectra of gelators **1** and **2** in D_2_O (self-aggregated state) and in CHCl_3_ (non-aggregated state). The absorption frequency for the C=O stretching band (amide I) in gels is always lower compared to that in CHCl_3_. The transmission bands of C=O stretching for the gel **1** and **2** in D_2_O appeared at 1660 and 1655 cm^−1^, respectively, which is characteristic of hydrogen bonded amide groups (Figure 5). Whereas the corresponding amide I stretching frequency at 1700 and 1703 cm^−1^ for **1** and **2** in CHCl_3_ demonstrates the existence of a non-hydrogen bonded amide group. Hydrogen bond formation is accompanied by a decrease in the bond order and hence the observed shift in the carbonyl stretching frequency to a lower value underlines the participation of intermolecular H-bonding in the gel state [37]. In addition, the N–H stretching frequency of amide for both gelators appeared at ≈3400 cm^−1^ in the gel state which was shifted to 3430 cm^−1^ in the non-aggregated form in CHCl_3_. This shift in the N–H stretching confirms the participation of the amide N-H in the intermolecular hydrogen bonding. Furthermore, the increase in intensity of the methylene scissoring vibration δ(CH_2_) band at ≈1460 cm^−1^ for both the gelators (Figure 5) in D_2_O indicates the high trans conformational packing of alkyl chain [38].

**Figure 6:**
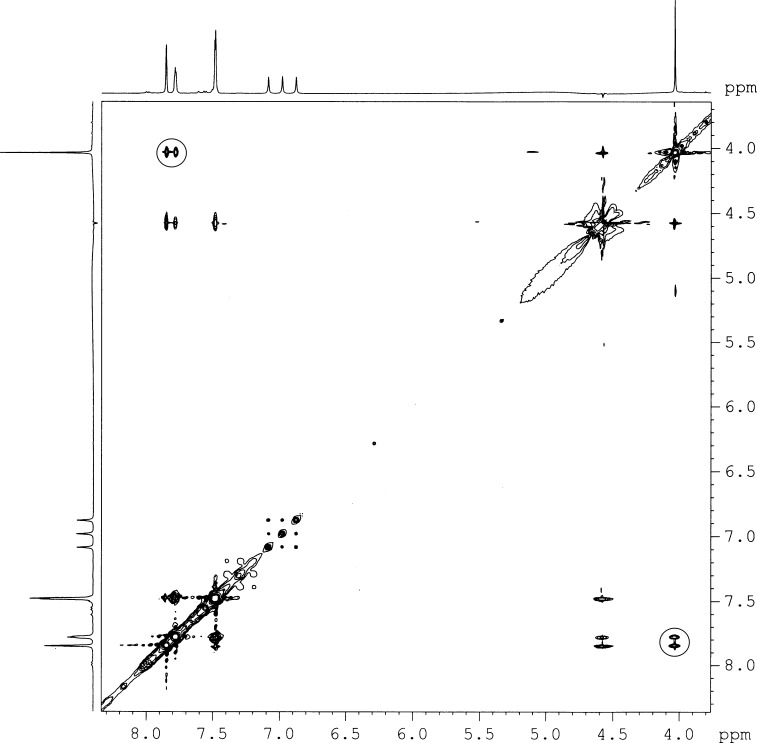
2D-NOESY spectra of **2** (2%, w/v) in DMSO-*d*_6_ with 70% water.

To establish further the intermolecular interaction between the gelators as noted above, we carried out 2D NOESY experiments for gelator **2** (2.0%, w/v) in the aggregated state in DMSO-*d*_6_ in the presence of 70% water and also in the non self-assembled state of the amphiphile in neat DMSO-*d*_6_. At 70% water content in DMSO-*d*_6_, off-diagonal cross peaks were observed between the aromatic rings and the methyl group on the quaternized nitrogen of pyridine (Figure 6). The presence of off-diagonal peaks in the aggregated form clearly indicates the existence of through space interaction between the neighboring gelator molecules which plays a crucial role in gelation. No such off-diagonal peak was observed for **2** in neat DMSO-*d*_6_ which is in accord with the absence of any kind of intermolecular interaction in the non-gelated state of the amphiphile.

**Figure 7:**
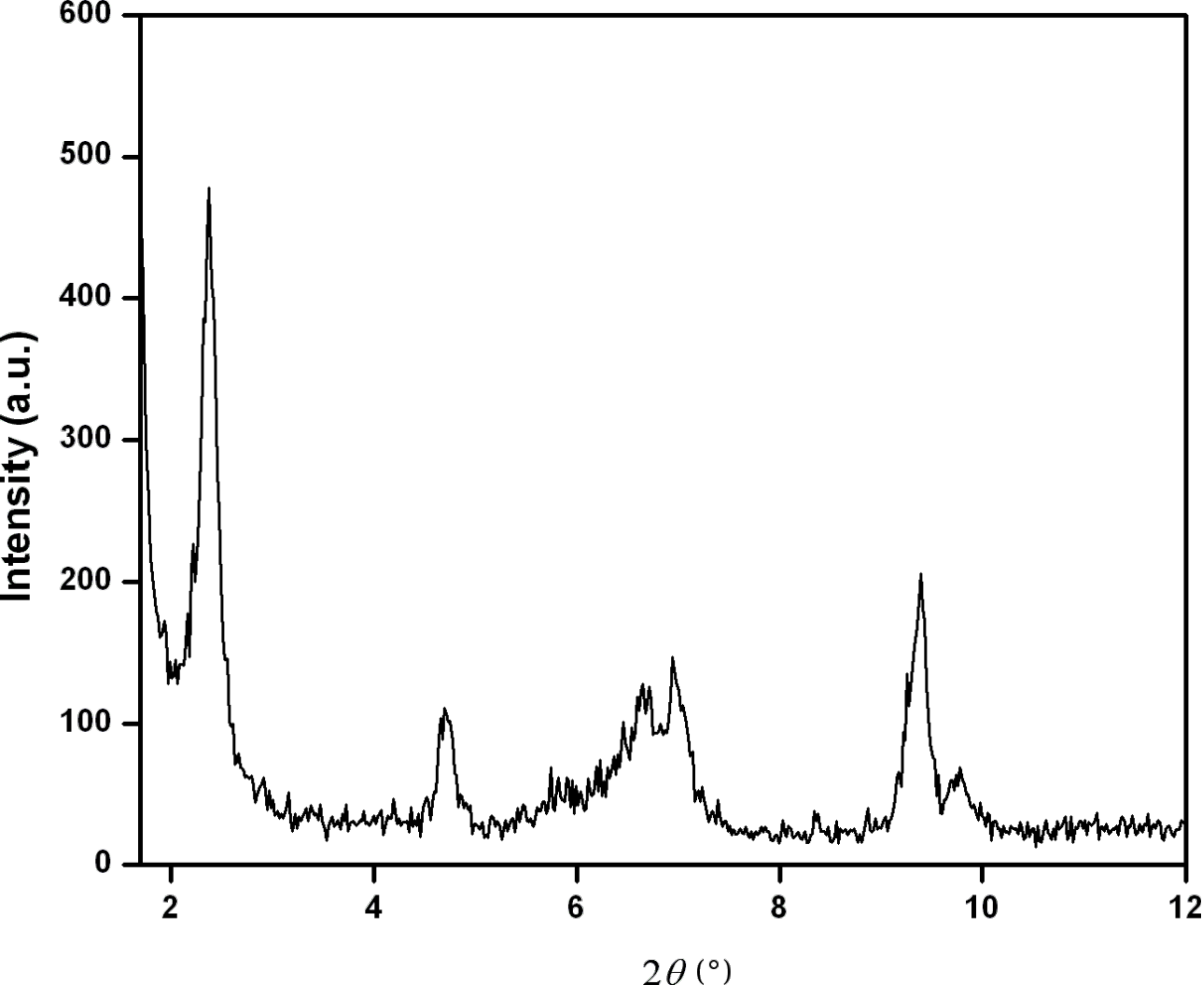
XRD diagram of the dried gel of **2**.

To investigate the molecular packing and orientation of the gelator molecules in the supramolecular self-assembled state, the xerogel of **2** was examined by X-ray diffraction (XRD). A sharp diffraction peak was obtained in the small angle region at 2θ = 2.37° which corresponds to a *d*-spacing of 3.71 nm indicating an ordered arrangement of the molecules in the gel state (Figure 7). The observed *d*-spacing was greater than the length of a single surfactant molecule, 2.6 nm (calculated using MOPAC AMI method, CS Chem Office) but smaller than twice the fully extended molecular length of gelator **2** (5.2 nm).

**Figure 8:**
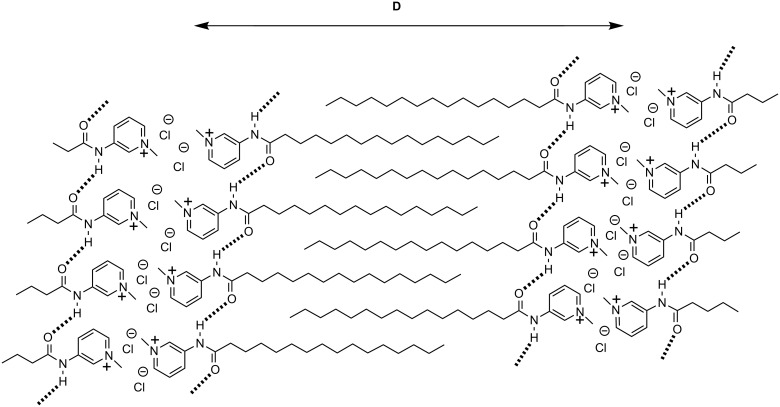
Schematic representation of the possible arrangement of molecules during hydrogelation of **2**.

Thus, on the basis of the aforementioned spectroscopic, microscopic studies as well as from the XRD results, it can be concluded that in the gelation process the amphiphiles are possibly forming repeating bilayers in which the molecules are connected by intermolecular hydrogen bonding and hydrophobic interaction. The probable interdigitated bilayer packing of the amphiphile **2** is represented in Figure 8 [39].

As noted earlier the pyridinium component is well known to impart antibacterial properties to a molecule [28–31]. Thus, we envisaged that it might be possible to develop inherently antibacterial soft matter based on amphiphilic pyridinium compounds. The antibacterial activities of both hydrogelating amphiphiles (**1** and **2**) were tested against two types of Gram-positive (*Bacillus subtilis* and *Micrococcus luteus*) and Gram-negative (*Escherichia coli* and *Klebsiella aerogenes*) bacteria. Minimum inhibitory concentrations (MIC), the lowest amphiphile concentration at which no viable bacterial cell is present, are presented in Table 1. Both **1** and **2** were found to be effective in killing bacteria with MIC values of 0.4–2.0 μg/mL for Gram-positive bacteria and 5.0–20.0 μg/mL for Gram-negative bacteria. However, **2** was found to have slightly better antibacterial activity than **1** with MIC values of only 0.4 μg/mL for Gram-positive *Micrococcus luteus* and 10 μg/mL for Gram-negative *Escherichia coli*. Interestingly, the pyridinium based amphiphilic hydrogelators showed antibacterial activity against both type of bacteria which is in contrast to the antibacterial activity of conventional quaternary cationic amphiphiles which are, in general, ineffective against Gram-negative bacteria. The positively charged amphiphiles are presumably adsorbed on the negatively charged cell membrane of microbes due to electrostatic interaction. This interaction is also entropically favorable as huge numbers of counterions are released. Next, the hydrophobic chain penetrates the hydrophobic cell membrane by ‘self-promoted’ transport resulting in release of the cytoplasmic constituents thus leading to the death of bacteria [29,40]. The pyridinium-based amphiphiles **1** and **2** are structurally different from those studied earlier [29] and their antibacterial activity is similar to that of the reported pyridinium compounds. Most importantly, the hydrogelation ability along with the inherent antibacterial properties of the present amphiphiles make them interesting scaffolds for biomedicinal applications.

**Table 1:** Antibacterial activities (MICs) of **1** and **2** in μg/mL.

Amphiphile	Gram-positive		Gram-negative	

	*B. subtilis*	*M. luteus*	*E. coli*	*K. aerogenes*

**1**	2.0	0.6	20.0	5.0
**2**	1.0	0.4	10.0	5.0

**Figure 9:**
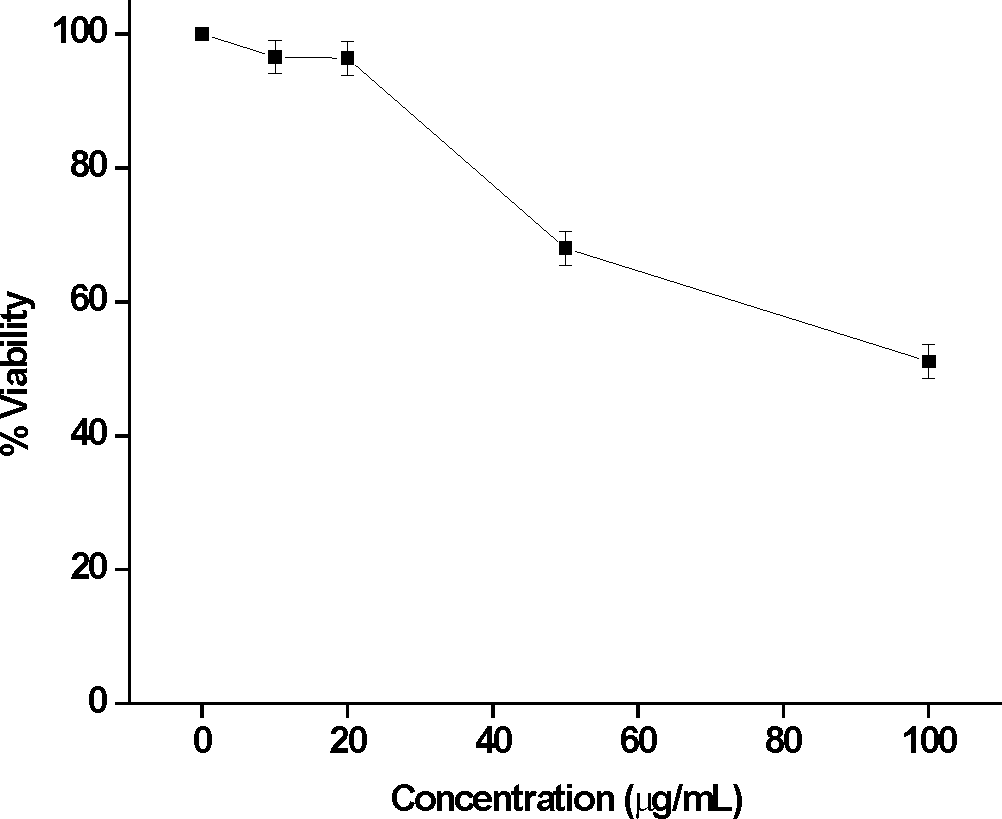
MTT assay based percent NIH3T3 cell viability as a function of concentration of amphiphile **2**.

Application of antibacterial biomaterials becomes more versatile and significant only when they are also non-toxic to living cells. Consequently, the cytotoxicity of amphiphile **2** (as a representative example) in NIH3T3 cells was investigated using MTT based assay. Encouragingly, the molecule showed more than 96% viability up to a concentration of 20 μg/mL. However, as the concentration of the amphiphile increased, viability towards the cell decreased. Nevertheless, even up to a concentration of 100 μg/mL, greater than 50% viability was noted (Figure 9). Thus, the cationic amphiphiles are potentially lethal to bacteria, but encouragingly viable to mammalian cells. Such cell selectivity may originate from the difference in the lipid composition as well as in the membrane potential gradient between the target prokaryotic and the non-target eukaryotic cell membranes [41–42].

## Conclusion

We have utilized a combination of a quaternary pyridinium unit and hydrophobic long chain to build a scaffold, which can gelate water. The routes adopted for the synthesis of such molecules were extremely simple. Systematic variations of the structure of the amphiphile reveal that minute architectural changes at molecular level influences the self-assembling mechanism of the gelation process. The major responsible factors for the gelation process were found to be non-covalent interactions such as π–π stacking and intermolecular hydrogen bonding. These cationic amphiphilic molecules exhibited antibacterial activity against both Gram-positive and Gram-negative bacteria and were found to be viable towards mammalian cells. The antibacterial activity conjugated with low cytotoxicity and water gelation ability makes this class of compound an attractive target for the development of antibacterial biomaterials.

## Experimental

### Materials

Myristic acid, palmitic acid, stearic acid and ethyl bromide were purchased from SRL, India. Thionyl chloride, 3-aminopyridine, methyl iodide were purchased from Spectrochem, India. D_2_O, DMSO-*d*_6_ and CDCl_3_ were obtained from Aldrich Chemical Co. Thin layer chromatography was performed on Merck pre-coated silica gel 60-F_254_ plates. All the material used in the cell culture study, such as Dulbecco’s Modified Eagles’ Medium (DMEM), heat inactivated fetal bovine serum (FBS), trypsin from porcine pancreas and MTT, were obtained from Sigma Aldrich Chemical Company. ^1^H NMR spectra were recorded on an AVANCE 300 MHz (BRUKER) spectrometer. Mass spectrometric data were acquired by the electron spray ionization (ESI) technique on a Q-Tof-micro Quadruple mass spectrometer (Micromass). Fluorescence and FTIR spectra were measured on a Varian Cary Eclipse luminescence spectrometer and a Perkin Elmer Spectrum 100 FTIR spectrometer, respectively.

### General synthetic procedure

#### Synthesis of amphiphiles 1–5

The acid (3 g) was refluxed with thionyl chloride (1 mL) for 4 h in an oil bath at 70 °C. The unreacted thionyl chloride was removed with a rotary-evaporator. The resulting compound (90% yield) was dissolved in dry dichloromethane (DCM) and then 3-aminopyridine (1.5 equiv dissolved in minimum quantity of dry DCM) added dropwise with stirring and ice cooling. The solution was stirred for 3–4 h, the DCM removed and the residue dissolved in ethyl acetate. The solution was washed with NaOH to remove excess acid and to convert the pyridinium salt to the free pyridine base. The organic layer was washed with brine until neutral. The ethyl acetate was then removed and the alkylated compound coupled through the amide linkage purified by column chromatography on 60–120 mesh silica gel with 1% methanol/chloroform mixture as eluent (75% yield). The compound thus obtained was stirred with methyl iodide (1.1 equiv) in dry DCM for 4–5 hours. After the reaction, the DCM was removed and the compound dissolved in ethyl acetate. The product was purified by column chromatography on 60–120 mesh silica gel with methanol/chloroform as eluent. The resulting iodide salt was subjected to ion exchange on Amberlite IRA-400 chloride resin to produce the pure chloride salt. The overall yield was ≈50–60%. Amphiphile **5** was quaternized with ethyl bromide (2 equiv) and stirred for 36 h. The reaction mixture was taken in chloroform and washed with aqueous sodium thiosulphate and brine solutions. The organic layer was evaporated (rotary evaporator) and finally purified by column chromatography on 60–120 mesh silica gel with methanol/chloroform as the eluent. In order to synthesize compound **4** the corresponding coupled compound was dissolved in methanol (minimum quantity) and HCl gas was passed through it. The precipitate formed was filtered and collected. General synthetic scheme for the preparation of all the amphiphiles (**1**–**5**) is shown in Scheme 1.

**Data for 1**: ^1^H NMR (300 MHz, CDCl_3_, 25 °C): δ = 0.88 (t, 3H), 1.26 (br, 28H), 1.66–1.75 (m, 2H), 2.65 (t, 2H), 4.45 (s, 3H), 7.88–7.93 (m, 1H), 8.44–8.46 (d, 1H), 9.24–9.27 (d, 1H), 9.85 (s, 1H), 10.79 (br, 1H) ppm; ESI-MS: *m/z* calcd for C_24_H_43_N_2_O (the quaternary ammonium ion, 100%) 375.3370; found 375.3335 [M]^+^; Elemental analysis calcd (%) for C_24_H_43_N_2_OCl: C, 70.12 H, 10.54; N, 6.81; found: C, 69.86; H, 10.31; N, 6.53.

**Data for 2**: ^1^H NMR (300 MHz, CDCl_3_, 25 °C): δ = 0.87 (t, 3H), 1.25 (br, 24H), 1.65–1.74 (m, 2H), 2.67 (t, 2H), 4.44 (s, 3H), 7.88–7.93 (m, 1H), 8.44–8.46 (d, 1H), 9.24–9.27 (d, 1H), 9.85 (s, 1H), 10.80 (br, 1H) ppm; ESI-MS: *m/z* calcd for C_22_H_39_N_2_O (the quaternary ammonium ion, 100%) 347.3057, found 347.2011 [M]^+^; Elemental analysis calcd (%) for C_22_H_39_N_2_OCl: C, 68.99; H, 10.26; N, 7.31; found: C, 69.21; H, 10.15; N, 7.23.

**Data for 3**: ^1^H NMR (300 MHz, CDCl_3_, 25 °C): δ = 0.87 (t, 3H), 1.24 (br, 20H), 1.66–1.70 (m, 2H), 2.68 (t, 2H), 4.56 (s, 3H), 7.80–7.82 (m, 1H), 8.11 (br, 1H), 9.42–9.43 (d, 1H), 10.05 (s, 1H), 12.55 (br, 1H) ppm; ESI-MS: *m/z* calcd for C_20_H_35_N_2_O (the quaternary ammonium ion, 100%) 319.2744; found 319.1093 [M]^+^; Elemental analysis calcd (%) for C_20_H_35_N_2_OCl: C, 67.67; H, 9.94; N, 7.89; found: C, 67.49; H, 10.02; N, 8.07.

**Data for 4**: ^1^H NMR (300 MHz, CDCl_3_, 25 °C): δ = 0.87 (t, 3H), 1.26 (br, 24H), 1.59 (br, 2H), 2.29 (t, 2H), 7.53 (m, 1H), 7.69–7.73 (m, 2H), 7.81 (s, 1H), 8.19 (br, 1H) ppm; ESI-MS: *m/z* calcd for C_21_H_37_N_2_O (the quaternary ammonium ion, 100%) 333.2900; found 333.1956 [M]^+^; Elemental analysis calcd (%) for C_21_H_37_N_2_OCl: C, 68.36; H, 10.11; N, 7.59; found: C, 68.43; H, 9.98; N, 7.37.

**Data for 5**: ^1^H NMR (300 MHz, CDCl_3_, 25 °C): δ = 0.87 (t, 3H), 1.25 (br, 24H), 1.41–1.45 (m, 2H), 1.70–1.75 (t, 3H), 2.61 (t, 2H), 4.61–4.64 (q, 2H), 7.85 (s, 1H), 8.38 (s, 1H), 9.18–9.20 (d, 1H), 9.8 (s, 1H), 11.28 (s, 1H) ppm; ESI-MS: *m/z* calcd for C_23_H_41_N_2_O (the quaternary ammonium ion, 100%): 361.3213; found 361.1093 [M]^+^; Elemental analysis calcd (%) for C_23_H_41_N_2_OCl: C, 69.58; H, 10.41; N, 7.06; found: C, 69.36; H, 10.26; N, 7.12.

#### Preparation of hydrogel

The required amount of the amphiphile was added in 1 mL water at pH = 7.0 to a screw-capped vial with an internal diameter of 10 mm and heated slowly until the solid had completely dissolved. The solution was then cooled to room temperature without any disturbance. After 1 h, formation of gel was confirmed by stable to inversion of the glass vial.

#### Microscopic studies

FESEM was performed on JEOL-6700F microscope. A piece of hydrogel was mounted on a glass slide and dried for few hours under vacuum before imaging. The morphology of the dried gel of compound **2** was also studied using AFM (Veeco, model AP0100) in the non-contact mode. A piece of gel was mounted on a silicon wafer and dried for a few hours under vacuum before imaging.

#### Fluorescence spectroscopy

The emission spectra of the compound **2** were recorded on Varian Cary Eclipse luminescence spectrometer in the concentration range from 0.01%, w/v to above MGC (3%, w/v). A super stock solution of **2** was prepared and diluted as required. The compound was excited at λ_ex_ = 330 nm and emission recorded between 340–550 nm. The excitation and emission slit widths were 5 nm and 5 nm, respectively.

#### FTIR measurements

FTIR measurements of the gelators **1** and **2** in CHCl_3_ solution and in D_2_O (gel state) were taken in a Perkin Elmer Spectrum 100 FTIR spectrometer using KBr and CaF_2_ windows, respectively with 1 mm Teflon spacers at their MGC.

#### NMR measurements

^1^H NMR and 2D-NOESY spectra were recorded on AVANCE 300MHz (BRUKER) spectrometer at 2%, w/v for **2** in DMSO-*d*_6_ and in water (70%) and DMSO-*d*_6_ (30%).

#### X-ray diffraction (XRD)

XRD measurements were taken with Seifert XRD 3000P diffractometer. The source was Cu Kα radiation (λ = 0.15406 nm) with a voltage and current of 40 kV and 30 mA, respectively. The gel was mounted on a glass slide and dried under vaccum. The xerogel was scanned from 2Θ = 1–40°.

#### Microorganisms and culture conditions

The in vitro antimicrobial activity of the cationic amphiphiles was investigated against representative Gram-positive and Gram-negative bacteria. Gram-positive bacteria used in the present study were *Bacillus subtilis* and *Micrococcus luteus*. Gram-negative bacteria investigated include *Escherichia coli* and *Klebsiella aerogenes*. Investigations of antibacterial activities were performed by the broth dilution method. The LB medium (tryptone (10 g), yeast extract (5 g) and NaCl (10 g) in 1 L sterile distilled water at pH 7.0) was used as the liquid medium in all antibacterial experiments. All the microbial strains were purchased from Institute of Microbial Technology, Chandigarh, India. The stock solutions of all the amphiphiles as well as the required dilutions were made in autoclaved sterile water. Freeze-dried ampoules of all bacterial strains were opened and a loopful of culture was spread to give single colonies on the respective solid LB agar media and incubated for 24 h at 37 °C. A representative single colony was picked up with a wire loop and was spread on an agar slant to give single colonies. The slants were incubated at 37 °C for the respective time. These incubated cultures of all the bacteria were diluted as required to give a working concentration in the range of 10^6^–10^9^ colony forming units (cfu)/mL before every experiment.

#### Antimicrobial studies

Minimum inhibitory concentrations (MICs) of hydrogelating amphiphiles **1** and **2** were estimated by both the broth dilution and the spread plate method. MIC was measured using a series of test tubes containing the amphiphiles (0.05–200 μg/mL) in 5 mL liquid medium. Diluted microbial culture was added to each test tube at identical concentrations to obtain the working concentration of bacteria: for *B*. *subtilis* 7.5 × 10^7^–1 × 10^8^ cfu/mL, for *M. luteus* 5 × 10^6^–7.5 × 10^6^ cfu/mL, for *E*. *coli* 3.75 × 10^7^–7.5 × 10^7^ cfu/mL, for *K. aerogenes* 9 × 10^7^–1.2 × 10^8^ cfu/mL. All the test tubes were then incubated at 37 °C for 24 h. The optical density of all the solutions was measured at 650 nm before and after incubation. Liquid medium containing microorganisms was used as a positive control. All the experiments were performed in triplicate and repeated twice.

#### Cell cultures

Mouse embryonic fibroblast cell NIH3T3 were obtained from National Center for Cell Science (NCCS), Pune and maintained in DMEM medium supplemented with 10% FBS, 100 mg/L streptomycin and 100 IU/mL penicillin. Cells were grown in a 25 mL cell culture flask and incubated at 37 °C in a humidified atmosphere of 5% CO_2_ to approximately 70–80% confluence. Media change was done after 2–3 days and subculture was performed every 7 days. After 7 days, media was removed to eliminate the dead cells. Next, the adherent cells were detached from the surface of the culture flask by trypsination. Cells were now in the exponential phase of growth for checking the viability of amphiphile **2**.

#### Cytotoxicity assay

The cytotoxicity of amphiphile **2** was assessed by the microculture MTT reduction assay as described in the literature. This assay is based on the reduction of a soluble tetrazolium salt by mitochondrial dehydrogenase of the viable cells to form an insoluble colored formazan product. The amount of formazan product formed can be measured spectrophotometrically after dissolution of the dye in DMSO. The activity of the enzyme and the amount of the formazan produced is proportional to the number of live cells. Reduction of the absorbance value can be attributed to the killing of the cells or inhibition of the cell proliferation by the molecule. 150 μL of cell solution were seeded (20,000 cells per well) in a 96-well microtiter plate for 18–24 h before the assay. A stock solution of the amphiphile **2** was prepared. Sequential dilution of this stock solution was carried out during the experiment to vary the concentrations of the amphiphile in the microtiter plate. The cells were incubated with the amphiphile solutions at different concentrations for 4 h at 37 °C under 5% CO_2_. Then, 15μL of MTT stock solution (5 mg/mL) in phosphate buffer saline was added to the above mixture and the cells were further incubated for another 4 h. The precipitated formazan was dissolved in DMSO and absorbance at 570 nm was measured using BioTek^®^ Elisa Reader. The number of surviving cells were expressed as percent viability = [A_570_ (treated cells)−background/A_570_(untreated cells)−background] × 100.
